# Akt1 sequentially phosphorylates p27^kip1 ^within a conserved but non-canonical region

**DOI:** 10.1186/1747-1028-1-11

**Published:** 2006-06-16

**Authors:** Lucas P Nacusi, Robert J Sheaff

**Affiliations:** 1 Department of Chemistry and Biochemistry, The University of Tulsa, Tulsa, Oklahoma 74104, USA; 2 Department of Biochemistry, molecular Biology and Biophysics, University of Minnesota Cancer Center, University of Minnesota, Minneapolis MN 55455, USA

## Abstract

**Background:**

p27^kip1 ^(p27) is a multifunctional protein implicated in regulation of cell cycling, signal transduction, and adhesion. Its activity is controlled in part by Phosphatylinositol-3-Kinase (PI3K)/Akt1 signaling, and disruption of this regulatory connection has been identified in human breast cancers. The serine/threonine protein kinase Akt1 directly phosphorylates p27, so identifying the modified residue(s) is essential for understanding how it regulates p27 function. Various amino acids have been suggested as potential targets, but recent attention has focused on threonine 157 (T157) because it is located in a putative Akt1 consensus site. However, T157 is not evolutionarily conserved between mouse and human. We therefore re-evaluated Akt1 phosphorylation of p27 using purified proteins and in cells.

**Results:**

Here we show purified Akt1 phosphorylates human and mouse p27 equally well. Phospho-peptide mapping indicates Akt1 targets multiple sites conserved in both species, while phospho-amino acid analysis identifies the targeted residues as serine rather than threonine. P27 deletion mutants localized these sites to the N-terminus, which contains the major p27 phosphorylation site in cells (serine 10). P27 phosphorylated by Akt1 was detected by a phospho-S10 specific antibody, confirming this serine was targeted. Akt1 failed to phosphorylate p27S10A despite evidence of a second site from mapping experiments. This surprising result suggested S10 phosphorylation might be required for targeting the second site. We tested this idea by replacing S10 with threonine, which as expected led to the appearance of phospho-threonine. Phospho-serine was still present, however, confirming Akt1 sequentially targets multiple serines in this region. We took two approaches in an attempt to explain why different residues were previously implicated. A kinetic analysis revealed a putative Akt1 binding site in the C-terminus, which may explain why mutations in this region affect p27 phosphorylation. Furthermore, commercially available recombinant Akt1 preparations exhibit striking differences in substrate specificity and site selectivity. To confirm S10 is a relevant site, we first showed that full-length wild type Akt1 purified from mammalian cells phosphorylates both human and mouse p27 on S10. Finally, we found that in cultured cells under physiologically relevant conditions such as oxidative stress or growth factor deprivation, endogenous Akt1 causes p27 accumulation by phosphorylating S10.

**Conclusion:**

Identifying where Akt1 phosphorylates p27 is essential for understanding its functional implications. We found that full-length wild type Akt1 – whether purified, transiently overexpressed in cells, or activated in response to cellular stress – phosphorylates p27 at S10, a noncanonical but evolutionarily conserved site known to regulate p27 activity and stability. Using recombinant Akt1 recapitulating this specificity, we showed modification of p27S10 also leads to phosphorylation of an adjacent serine. These results integrate PI3K/Akt1 signaling in response to stress with p27 regulation through its major phosphorylation site in cells, and thus identify new avenues for understanding p27 deregulation in human cancers.

## Background

Information transmitted by signaling pathways determines whether a cell continues the proliferative cycle or adopts an alternative fate. This decision requires regulating Cyclin Dependent Kinases (CDKs), which are activated by phosphorylation and temporal association with a unique cyclin subunit (D, E or A type in G1/S phase) [reviewed in [1]]. Two families of CDK inhibitory proteins (CKIs) have been identified: INK4 proteins (p15, p16, p18 and p19) specifically inhibit cyclin D-CDK4/6, while Cip/Kip proteins (p21, p27, and p57) are considered more broad-spectrum inhibitors of cyclin D, E, and A CDK complexes [2,3].

P27 regulation is particularly important because it functions as a tumor suppressor that is often disrupted in human cancers, usually by compromising its stability and/or location [4-7]. Consistent with this view mice without p27 develop pituitary tumors and display increased susceptibility to carcinogens [8-10]. Mice lacking a single copy of the p27 gene are still hypersensitive to carcinogens, illustrating the importance of precisely controlling its levels and activity [11,12]. Despite these observations the role of p27 in tumorigenesis remains enigmatic due to its multifunctionality. Although first characterized as a CDK inhibitor that negatively regulates cell cycle progression [13-16], p27 also possesses CDK-independent functions such as inhibiting the adaptor protein GRB2 (to regulate signaling) or targeting RhoA (to regulate adhesion) [17-19]. Disrupting these p27 activities could also contribute to the disease state, especially given recent evidence cancer cells do not necessarily require hyperactive CDKs [20,21].

**Figure 1:**
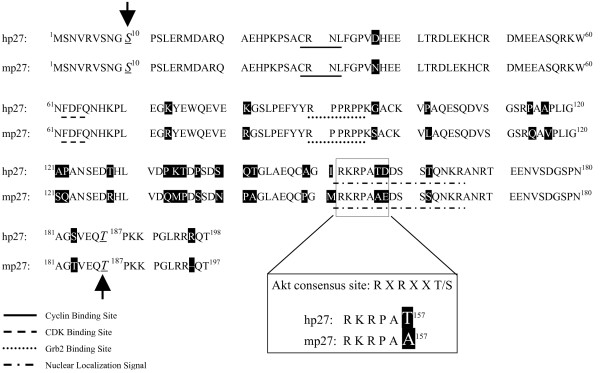
**p27 is highly conserved between human and mouse**. Comparison of human and mouse p27 sequences reveals they are 92% identical. Highlighted amino acids show absolute differences. Underlined amino acids indicate functional motifs: Cyclin E (RXL), CDK2 (FNF), Grb2 (PXXP) binding sites and the bipartite Nuclear Localization Signal (aa 152–166). Solid arrows show conserved phosphorylation sites. Blow up of Akt1 consensus site shows T157 is not maintained between species.

P27 multifunctionality also likely explains its complex regulation. The protein contains distinct cyclin and CDK binding sites at its N-terminus, a C-terminus motif responsible for interacting with RhoA, a proline rich domain for binding GRB2 (aa 90–95), and a bipartite nuclear localization signal (NLS; aa 162–176) [3,19,22,23] (Figure 1). Posttranslational modifications like phosphorylation regulate p27 activity by modulating its levels, location, and/or association with binding partners. Cyclin E-CDK2 phosphorylates p27 at threonine 187 (T187) in late G1 phase, initiating p27 ubiquitination and proteasomal degradation [24-27]. Serine 10 (S10) appears to be the major site of p27 phosphorylation in cells, and is targeted by several kinases including Map kinase, a recently identified mitogen responsive kinase called hKIS, and possibly Akt1 [28-30]. S10 modification stabilizes p27 and promotes its nuclear export via the carrier protein CRM1, thereby alleviating cyclin-CDK inhibition and perhaps initiating p27 cytoplasmic functions [31,32]. A better understanding of what kinase(s) target S10 would provide much needed insight into the physiological relevance of modifying this residue.

The PI3K/Akt1 pathway plays a key role in cell survival and proliferation by modulating gene expression, protein synthesis, and cell cycle machinery [33]. PI3K regulates p27 function via a multi-pronged effort that includes altering p27 transcription, protein levels, and location [34,35]. Akt1 contributes to this process by phosphorylating and inhibiting Forkhead transcription factors that promote p27 transcription [36]. In addition, Akt1 directly phosphorylates p27 in order to alter its levels and/or location in the cell [30,37-42]. Thus, inappropriate PI3K/Akt1 activation could contribute to cellular transformation in part by deregulating p27 [35]. Identifying the p27 residue(s) phosphorylated by Akt1 is essential for determining their regulatory significance. Numerous sites have been proposed, but recent attention has focused on threonine 157 (T157). This residue is located in an Akt1 consensus sequence "RKRPAT^157^" within the p27 NLS. Recombinant Akt1 failed to phosphorylate a p27 mutant lacking this site, and in some breast cancer cell lines Akt1 hyperactivation altered p27 location [39-42].

Important p27 phosphorylation sites are usually conserved among species (e.g. T187 and S10), but T157 is an alanine in the mouse. This observation prompted us to re-evaluate the site(s) targeted by Akt1. We now show purified Akt1 phosphorylates mouse and human p27 equally well. Phospho-peptide and phospho-amino acid analysis revealed Akt1 modifies neighboring serine residues that are conserved among species. Analysis of p27 deletion mutants localized these residues to the N-terminus 1–86 amino acids, which contain the well known S10 phosphorylation site. We confirmed Akt1 phosphorylates S10 by western blot using an antibody specific for phospho-S10. Surprisingly, Akt1 failed to phosphorylate p27S10A despite mapping evidence indicating existence of an additional site. This suggested S10 phosphorylation might be a pre-requisite for its modification. We confirmed this idea using a p27 mutant containing threonine in place of S10, which now resulted in both radiolabeled threonine and serine. Our data indicate Akt1 sequentially phosphorylates multiple serine residues within a conserved but non-canonical region, one of which (S10) has already been identified as a major regulator of p27 function. These results were confirmed in cultured cells where co-expressing Akt1 with p27 leads to an increase in S10 phosphorylation. Finally, we show that stressing tissue culture cells with oxidative insult or growth factor deprivation causes an Akt-dependent increase in p27S10 phosphorylation.

## Results

### Akt1 phosphorylates both human and mouse p27

**Figure 2:**
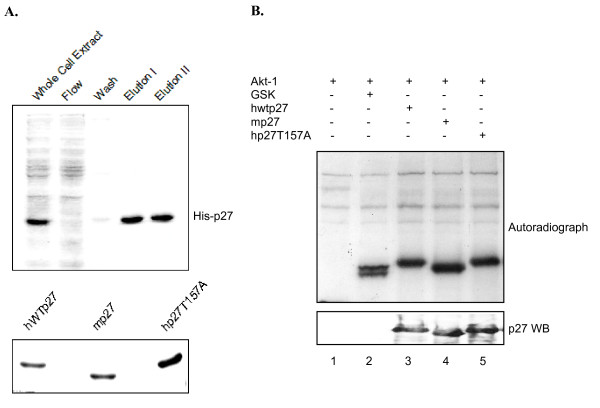
**Akt1 phosphorylates human and mouse p27**. *A*, Purification of His-tagged p27 from E. coli. Upper panel: Aliquots from indicated steps of the purification protocol (see Methods) were separated by SDS-PAGE and visualized by Coomassie blue staining. Bottom panel: Indicated forms of His-tagged p27 were purified as described in Methods and their concentration compared by SDS-PAGE followed by Coomassie staining. *B*, Constitutively active Akt1 phosphorylates p27 lacking T157. An in vitro kinase assay was performed as described in the Methods by incubating commercial recombinant Akt1(prep1) (see Methods) and [^32^P]ATP with the indicated substrates. Samples were then separated by SDS-PAGE and visualized by autoradiography. Lane 1 is a negative control lacking Akt1 substrate, while lane 2 shows Akt1 phosphorylates its well-known substrate GSK. Lane 3–5 show Akt1 phosphorylates human wild type p27, mouse p27, and hp27T157A equally well.

Numerous reports indicate Akt1 directly phosphorylates human p27 [30,37-42]. Several potential sites have been suggested, based mainly on the failure of Akt1 to phosphorylate purified p27 containing site specific mutations [30,39-42]. A threonine located at position 157 of human p27 recently emerged as a leading candidate, in part because it resides within an Akt1 consensus sequence (RXRXXT^157^). If this residue is targeted then Akt1 should not radiolabel mouse p27 (which contains alanine at position 157) or human p27 in which T157 has been mutated to alanine. To test these predictions we established an *in vitro *kinase assay similar to that employed by other groups [39-41]. It utilizes recombinant, constitutively active Akt1 obtained from a commercial supplier (see Methods), and His-tagged forms of p27 purified from E. coli. The upper panel of Figure 2A is a Coomassie stained gel showing a typical purification profile of human His-tagged p27, while the lower panel compares the purified forms of p27 utilized in subsequent assays. Mouse p27 is easily identified because it migrates slightly faster than the human version. Purified Akt1 was incubated with its well characterized substrate GSK (positive control) or the different p27s in the presence of [^32^P]-γ-ATP. Consistent with earlier reports recombinant Akt1 phosphorylated both GSK and human p27 (Figure 2B; lanes 2 and 3). However, Akt1 also phosphorylated mouse p27 and p27T157A to a similar extent (Figure 2B; lanes 4 and 5), suggesting Akt1 targets a site(s) other than T157.

### Akt1 phosphorylates p27 at multiple serines

**Figure 3:**
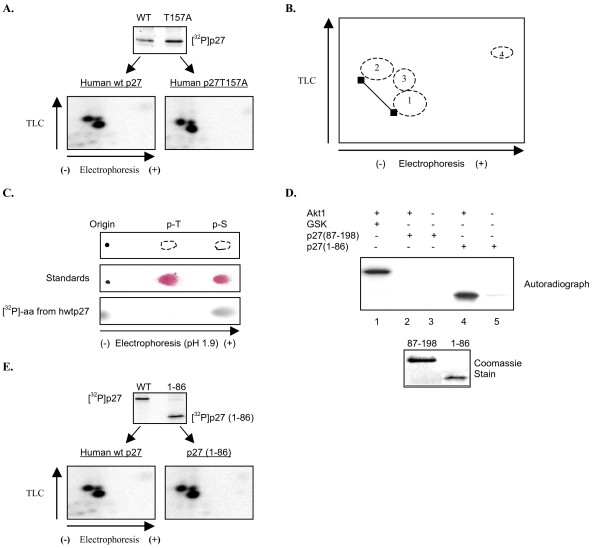
**Akt1 phosphorylates p27 at multiple serines within the N-terminus**. *A*, Phospho-peptide maps comparing hwtp27 to hp27T157A. The kinase assay was performed as described with indicated forms of p27. After SDS/PAGE separation (upper panel) radiolabeled p27 was subjected to two-dimensional phospho-peptide analysis (lower panel) (See Methods for details). Direction of electrophoresis and chromatography are indicated by arrows. *B*, Schematic representation of phospho-peptide pattern. Ellipses 1–3 correspond to phospho-peptides in 3A, while number 4 represents [^32^P]. *C*, Akt1 phosphorylates p27 at serines. Radiolabeled human wtp27 was hydrolyzed to constituent amino acids and then separated by electrophoresis on a TLC plate as described in Methods. Top panel: scheme representing migration of the phospho-amino acid standards (phospho-S and phospho-T). Middle panel: Phospho-amino acid standards separated by electrophoresis and visualized by 0.25% ninhydrin (see Methods). Bottom panel: phospho-amino acid analysis of [^32^P]hwtp27 visualized by phosphoimager. *D*, Akt1 phosphorylates p27(1–86). Kinase assay was performed as described with His-purified p27(1–86) and p27(87–198). Samples were separated by SDS/PAGE and visualized by autoradiography. Commassie staining shows the levels of deletion mutants were similar. *E*, Phospho-peptide maps comparing hwtp27 to hp27(1–86). Radiolabeled hwtp27 and p27(1–86) were analyzed as in *A*.

To determine if Akt1 phosphorylates p27 at multiple sites, we generated a phospho-peptide map by digesting radiolabeled p27 with trypsin and separating the resulting peptides in two dimensions. A single site should correspond to a single radiolabeled peptide. Conversely, if mutating T157 to alanine alters specificity then the phospho-peptide pattern should change. We therefore compared phospho-peptide maps for human wild type p27 and the p27T157A mutant. After the kinase assay [^32^P]p27 was separated by SDS-PAGE and transferred to PDVF membrane. It was then excised and subjected to tryptic digestion. Resulting peptides were separated by high voltage electrophoresis followed by ascending chromatography as described in Methods. The phospho-peptide maps of wtp27 and p27T157A are identical, indicating the same site(s) are targeted (Figure 3A). The maps clearly show three prominent radiolabeled spots of varying intensity. Their orientation is depicted in Figure 3B, while the fourth spot is inorganic phosphate and thus serves as an internal control. Phospho-peptides 1 and 2 are lying in a diagonal characteristic of phospho-isomers (variable number of phosphates on the same peptide) [43]. Identity of the less intense spot 3 is unclear. It may result from partial digestion or phosphorylation of a less efficiently targeted residue located on a different peptide.

Six tryptic peptides from p27 contain multiple serine and/or threonine residues (Table 1). We therefore performed a phospho-amino acid analysis to determine whether Akt1 targets serine, threonine or both. Surprisingly, Akt1 phosphorylated hwtp27 predominantly on serine (Fig 3C). Because multiple serine residues are located on peptides throughout p27, we utilized deletion mutants to determine if Akt1 phosphorylates the N- or C-terminus. Recombinant Akt1 phosphorylated p27(1–86) but not p27(87–198) (Figure 3D; compare lanes 2 and 4). To rule out the possibility Akt1 specificity was altered by the deletion, we performed phospho-peptide analysis of (1–86) and full length p27. The maps are identical (Figure 3E), indicating Akt1 phosphorylates multiple serines located between amino acids 1–86.

**Table 1:** Tryptic peptides for Human p27^Kip1^

*Peptides*	*Position*
MSNVR	1–5
VSNGSPSLER	6–15
QAEHPKPSACR	20–30
DMEEASQR	51–58
GSLPEFYYRPPRPPK	82–96
VPAQESQDVSGSR	101–113
PAAPLIGAPANSEDTHLVDPK	114–134
TDPSDSQTGLAEQCAGIR	135–152
PATDDSSTQNK	155–165
TEENVSDGSPNAGSVEQTPK	170–189
QT	197–198

**Figure 4:**
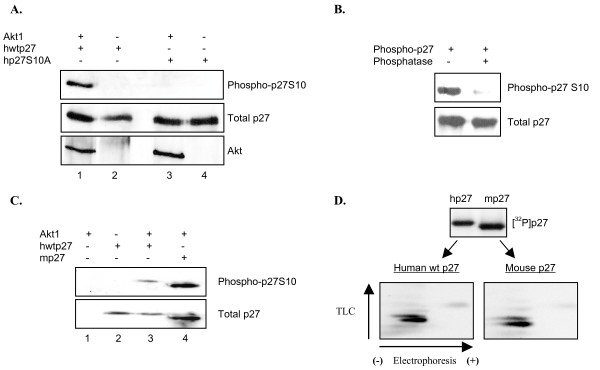
**Akt1 phosphorylates p27S10**. *A*, Determination of S10 phosphorylation by specific antibody. Recombinant Akt1 was incubated with the indicated forms of His-tagged p27 in the presence of non-labeled ATP. Samples were separated by SDS/PAGE and analyzed by western blot with the indicated antibodies. *B*, Alkaline phosphatase abolishes recognition of phosphorylated p27. hwtp27 was phosphorylated as in *A *and treated with or without alkaline phosphatase. Samples were then analyzed by western blot with the indicated antibodies. *C*, Akt1 phosphorylates serine 10 in mouse p27. Akt1 was incubated alone or with the indicated forms of p27 in the presence of non-labeled ATP. Total and phospho-S10 levels were analyzed by western blot as indicated. *D*, Phospho-peptide maps of human and mouse p27. Radiolabeled samples were analyzed as described in Figure 3A.

### Akt1 phosphorylates serine 10

The predicted tryptic digest shows only one N-terminus peptide containing two serines (Table 1; amino acids 6–15). Our attention was immediately drawn to S10 – despite the fact it is not within an Akt1 consensus motif – because it is a well known, physiologically relevant p27 phosphorylation site. To determine if S10 is one of the sites targeted by Akt1 we utilized a commercially available antibody directed against p27 phosphorylated at this location. This antibody clearly recognizes p27 only after incubation with Akt1 in a kinase reaction (Figure 4A). In contrast, it failed to recognize p27S10A whether or not Akt1 was present. To further confirm that the S10 antibody is recognizing phosphorylated p27, products of the kinase reaction were incubated alone or in the presence of alkaline phosphatase (Figure 4B). As expected, the signal was significantly diminished after phosphate removal. Serine 10 and surrounding amino acids are conserved in both mouse and human p27 (Figure 1), and we confirmed Akt1 targets S10 in mouse p27 using the same methodology (Figure 4C; lanes 3 and 4). Furthermore, phospho-peptide maps of mouse p27 were identical to that of the human protein, confirming the same sites are phosphorylated in both species (Figure 4D).

### P27S10 phosphorylation is required to target a second site

**Figure 5:**
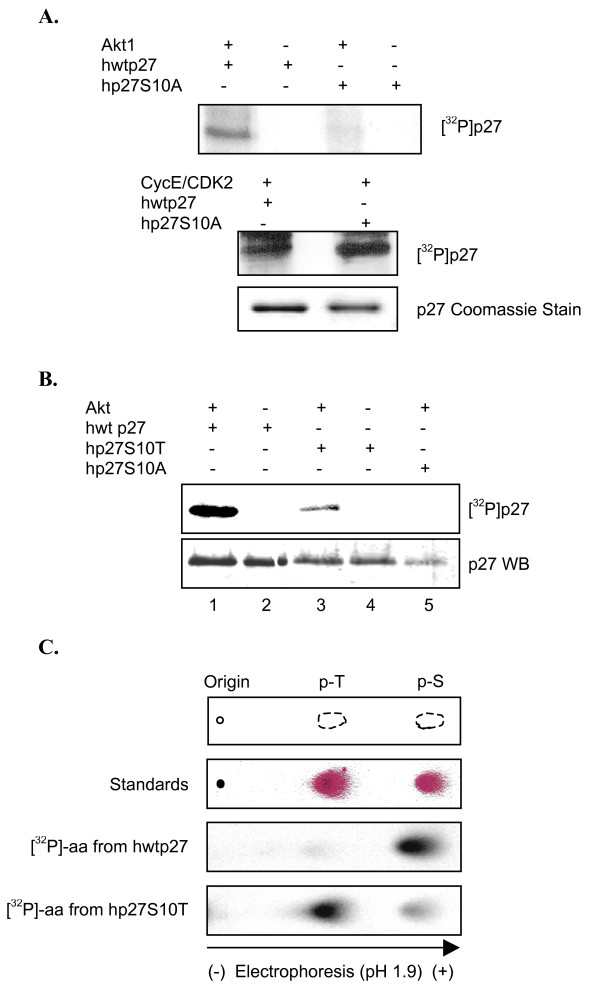
**Akt1 sequentially phosphorylates p27 at S10 and a neighboring serine**. *A*, Akt1 does not phosphorylate hp27S10A. Upper panel: Recombinant Akt1 was incubated in a kinase reaction with the indicated forms of p27 as described. Samples were separated by SDS/PAGE and visualized by autoradiography. Bottom panel: His-tagged cyclin-E-CDK2 was purified from transfected HEK293 cells (see Methods), then incubated with indicated forms of p27 in the presence of [^32^P]-γ-ATP. Samples were separated by SDS-PAGE and visualized by autoradiography. Commassie staining shows equal amounts of hwtp27 and p27S10A were present in the reactions. *B*, Akt1 phosphorylates hp27S10T. Kinase reaction was performed with recombinant Akt1 and indicated substrates. Upper panel: autoradiograph showing phosphorylated p27. Bottom panel: western blot showing similar levels of p27 in the reaction. *C*, Phospho-amino acid analysis comparing hwtp27 and hp27S10T. Kinase reaction described in *B *was repeated and radiolabeled p27 was subjected to PAA as in Figure 3C. Top panel: scheme representing migration of phospho-amino acid standards. Second panel: Phospho-amino acid standards separated by electrophoresis and visualized by 0.25% ninhydrin. Third panel: phospho-amino acid analysis of radiolabeled hwtp27. Bottom panel: phospho-amino acid analysis of hp27S10T. Radiolabeled peptides and amino acids were detected by phosphoimager.

Phospho-peptide mapping suggests Akt1 targets multiple sites (Figure 3A). Thus, we expected that mutating S10 to alanine would not completely ablate p27 phosphorylation. Surprisingly, however, Akt1 failed to radiolabel hp27S10A (Figure 5A, top panel). To rule out the possibility p27S10A was misfolded or otherwise compromised, a kinase reaction was carried out using cyclin E-CDK2 purified from HEK293 cells as described in Methods. Both hp27 and hp27S10A were phosphorylated equally well by cyclin E-CDK2 (Figure 5A, middle panel) suggesting hp27S10A structure is not dramatically disrupted. These results not only confirm Akt1 targets S10, but also suggest its modification is required for targeting another serine.

We directly tested this idea by taking advantage of the fact Akt1 is a serine/threonine kinase. If S10 alone is phosphorylated then converting it to threonine should only generate radiolabeled phospho-threonine. However, if an additional serine is also targeted both phospho-threonine and phospho-serine should be present. We generated a p27S10T mutant by site-directed mutagenesis. Akt1 still phosphorylated hp27S10T, albeit more weakly than wild type p27 (Figure 5B, compare lanes 1 and 3). Nevertheless, the amounts were sufficient for phospho-amino acid analysis. As shown previously Akt1 phosphorylated wild type p27 almost exclusively on serines (Figure 5C, third panel). In contrast, p27S10T clearly shows both phospho-serine and phospho-threonine (Figure 5C, bottom panel). These results provide further evidence Akt1 targets S10, and reveal existence of a second modified serine. Because p27S10A is not radiolabeled by Akt (see Figure 5A and *B*), we conclude that modification of the second site is dependent on first phosphorylating S10. This scenario could also explain why the second site appears to be phosphorylated less efficiently.

### Kinetic analysis of p27 phosphorylation

**Figure 6:**
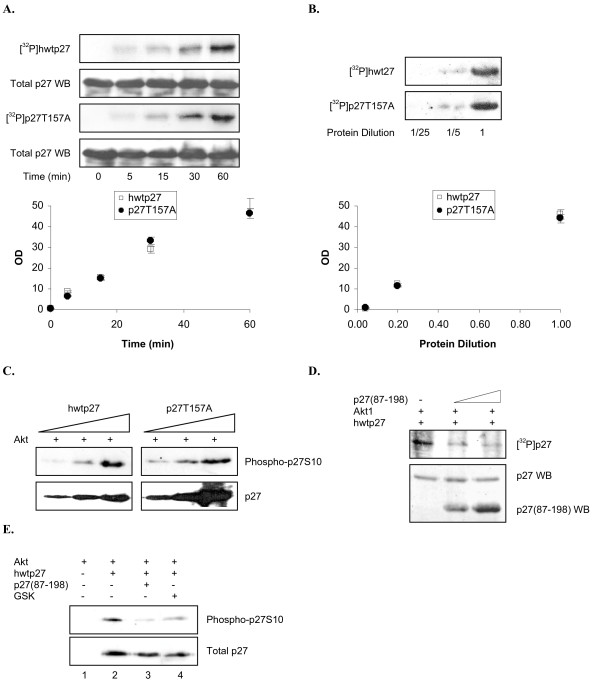
**Kinetic analysis of p27 phosphorylation**. *A*, Time course of p27 phosphorylation. Kinase reaction was performed as described with hwtp27 or p27T157A and stopped at indicated times. Phosphorylation is shown by autoradiograph and total p27 by western blot. The amount of [^32^P] incorporated was measured by densitometry, expressed as OD, and plotted *vs*. reaction time. [^32^P] incorporation was normalized against the highest value arbitrarily set at 1. *B*, Phosphorylation rate as a function of p27 concentration. Indicated dilutions of hwtp27 and p27T157A were utilized in the kinase reaction. Autoradiographs show [^32^P] incorporation. Graph shows radiolabeled p27 quantitated as in *A vs*. protein dilution. *C*, Rate of S10 phosphorylation. Indicated forms of His-tagged p27 were incubated with Akt1 and non-labeled ATP. Samples were separated by SDS-PAGE and visualized by western blotting with the indicated antibodies. *D*, p27(87–198) inhibits Akt1 phosphorylation of p27. Kinase reaction was performed with hwtp27, Akt1 and [^32^P]-γ-ATP in the presence of indicated amounts of p27(87–198). Upper panel: autoradiograph. Bottom panel: western blot showing levels of full length and C-terminus of p27. *E*, p27(87–198) inhibits Akt1 phosphorylation of p27S10. Kinase reaction was performed as described in *D *with non-labeled ATP in the presence of p27(87–198) or GSK. S10 phosphorylation was determined by western blot analysis.

Given our extensive evidence Akt1 phosphorylates multiple serines within the N-terminus, it is unclear why previous studies implicated other residues. A limitation of identifying phosphorylation sites solely by mutational analysis is the potential for indirect effects (e.g. overall protein structure can be compromised, or an enzyme binding site can be ablated). In the case of p27 several of the previously identified Akt1 targets localize to the C-terminus, so their mutation might disrupt an Akt1 binding region that alters phosphorylation of N-terminus serines. To test this idea we analyzed kinetics of hwtp27 and p27T157A phosphorylation. Figure 6A shows the rate of p27T157A phosphorylation is similar to that of wild type. Since excess p27 could obscure differences in binding affinity that affect the rate, we repeated the analysis at various p27 concentrations. Rates were again similar, suggesting mutation of T157 does not dramatically affect Akt1 association with p27 (Figure 6B). Similar results were obtained when looking specifically at S10 phosphorylation using the phospho-p27S10 antibody (Figure 6C).

Even though mutating T157 to alanine had no obvious effect on p27 phosphorylation, other residues in the C-terminus might still be involved in Akt1 binding. We addressed this question by determining if p27(87–198) can inhibit phosphorylation of the full length protein. Recombinant Akt1 was incubated with [^32^P]-γ-ATP and full length p27 in the presence of increasing C-terminus. Figure 6D shows phosphorylation of hwtp27 was diminished when p27(87–198) was present. Similar results were obtained when the outcome was measured using the phospho-specific S10 antibody (Figure 6E). Thus, mutations in the p27 C-terminus could conceivably compromise an Akt1 binding site and affect phosphorylation at N-terminal residues. Although we were unable to demonstrate this consequence when T157 was mutated in our system, alteration of this site might affect p27 phosphorylation under different reaction conditions.

### Purified preparations of recombinant Akt1 show different specificities

**Figure 7:**
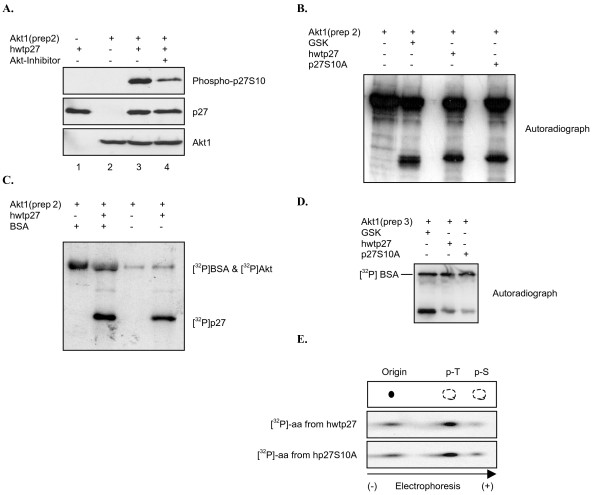
**Commercial recombinant Akt preparations show different specificity**. *A*, S10 phosphorylation by recombinant Akt1(prep2; see Methods). Recombinant Akt1(prep2) was incubated with His-tagged p27 in the presence of non-labeled ATP. Akt inhibitor was added where indicated. Samples were separated by SDS/PAGE and analyzed by western blot with the indicated antibodies. *B*, Radiolabeled phosphate incorporation by Akt1(prep2). Reaction was carried out as described in Methods. Autoradiograph shows phosphorylation of GSK, hwtp27 and p27S10A. *C*, Akt1(prep2) lacks specificity. Kinase reaction as described in *B *was performed in the presence or absence or bovine serum albumin (BSA). Autoradiograph shows Akt1(prep2) phosphorylates BSA as well as p27. *D*, Radiolabeled phosphate incorporation by Akt1(prep3). Kinase reaction performed as in *B *with recombinant Akt1(prep3; see Methods). *E*, Phospho-amino acid analysis of hwtp27 and p27S10A phosphorylated by Akt1(prep3). Phosphorylated p27 was subjected to PAA as described in Methods. Upper panel: schematic representation of phospho-amino acid migration. Middle panel: PAA corresponding to hwtp27. Bottom panel: PAA corresponding to p27S10A.

A survey of the literature revealed that putative Akt1 phosphorylation sites in p27 have been identified using similar in vitro kinase assays. However, in most cases the purified recombinant Akt1 was obtained from various commercial suppliers, and how it was activated, produced, and purified is different. We therefore analyzed activity of additional commercially available Akt1 preparations to determine if they yielded similar results. A carboxy-terminal His-tagged Akt1 (Akt1-prep2) phosphorylated p27S10 as indicated by the phospho-specific S10 antibody (Figure 7A). This reaction was largely inhibited by an Akt inhibitor, suggesting S10 was modified by Akt rather than a contaminating kinase. However, when we repeated the reaction using [^32^P]-γ-ATP, we were surprised to discover that in contrast to our earlier results Akt1-prep2 also phosphorylated p27S10A (Figure 7B). We noted that Akt1-prep2 appeared to lack specificity, as it efficiently phosphorylated BSA to the same extent as p27 (Figure 7C). Similar results were obtained with another recombinant Akt1 sample (Akt1-prep3: truncated, purified from insect cells and activated using PDK1) (Figure 7D), and in this case threonine in p27 was the main target (Figure 7E). These observations could explain why different p27 residues have been identified as targets for Akt1. Our initial analysis was also carried out using purified active Akt1 obtained from a commercial supplier. We were fortunate, however, in that this preparation displays greater specificity for physiologically relevant substrates and does not phosphorylate BSA (Figure 2B). Although these observations suggest S10 is likely a physiologically relevant site, to further resolve this issue we analyzed p27 phosphorylation by wild type full length Akt1.

### Full length Akt1 from mammalian cells phosphorylates serine 10 in vitro

**Figure 8:**
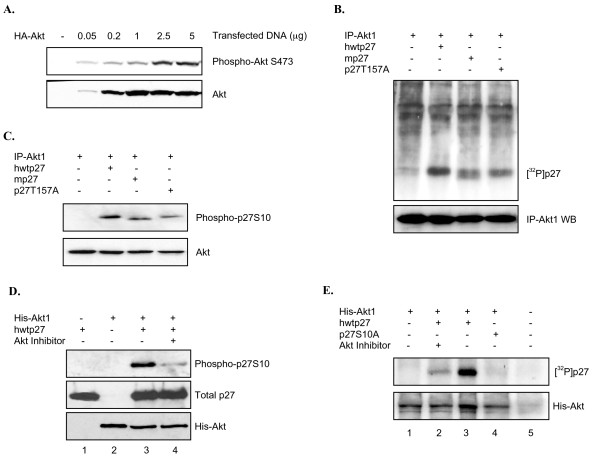
**Full length Akt1 phosphorylates p27 at serine 10 in vitro and in cells**. *A*, Expression and activation of Akt1 in HEK293 cells. Increasing amounts of HA-tagged wtAkt1 were transfected in HEK293 cells as described in the Methods. After 36 hours active and total levels of Akt1 were determined by western blot analysis. *B*, Full length wild type Akt1 phosphorylates p27. Overexpressed HA-Akt1 was purified by immunoprecipitation (IP-Akt1) and incubated alone or with indicated forms of His-p27 in the presence of [^32^p]-γ-ATP as described in Methods. Autoradiograph shows full length Akt1 phosphorylates human and mouse p27 as well as the hp27T157A mutant. *C*, Wild type Akt1 targets S10. The kinase reaction was performed by incubating IP-Akt1 with non-radiolabeled ATP and indicated substrates. Western blot shows full length Akt phosphorylates S10. *D*, Specific Akt inhibitor blocks S10 phosphorylation. A kinase reaction was performed as in *C *incubating full length His-purified Akt1 with p27 in the presence or absence of Akt inhibitor. S10 phosphorylation was determined by phospho-specific antibody. *E*, Full length Akt1 fails to phosphorylate hp27S10A. Full length His-Akt1 was incubated in a kinase reaction with the indicated forms of p27 in the presence of radiolabeled ATP. Samples were separated by SDS/PAGE and visualized by autoradiography. Akt inhibitor was added where indicated.

We first repeated the in vitro kinase assay using full length wild type Akt1 purified from mammalian cells. HEK293 cells were transfected with increasing amounts of a plasmid containing HA-tagged Akt1 under control of the CMV promoter. Equal amounts of cell extract (as determined by Bradford assay) were then subjected to western blot analysis. Figure 8A shows HA-tagged Akt1 was overexpressed and activated in HEK293 cells. We therefore repeated the transfection and purified HA-Akt1 by immunoprecipitation with an HA antibody (IP-Akt1). Figure 8B shows full length Akt1 phosphorylates hwtp27, mp27 and p27T157A, similar to results obtained in our initial analysis with recombinant enzyme (Figure 2B). Furthermore, in all cases full length wild type Akt1 phosphorylated S10 as indicated by western blotting with the phospho-specific antibody (Figure 8C). To rule out the possibility another kinase co-precipitated with HA-Akt or was nonspecifically pulled down by the antibody, we overexpressed His-tagged Akt1 in HEK293 cells, purified it on a Nickel column, and performed a kinase assay with non-labeled ATP. His-Akt phosphorylated p27 at S10 as determined by phospho-specific S10 antibody (Figure 8D, lane 3) and the reaction was inhibited by an Akt inhibitor (Figure 8D, compare lanes 3 and 4). To confirm S10 is the major site we looked for incorporation of radiolabeled phosphate onto p27S10A. Figure 8E shows His-Akt phosphorylates hwtp27 and that this reaction is blocked by Akt inhibitor (compare lanes 3 and 2). In contrast, His-Akt completely fails to phosphorylate p27S10A (lane 4).

### Stress activated Akt phosphorylates p27S10 in cultured cells

**Figure 9:**
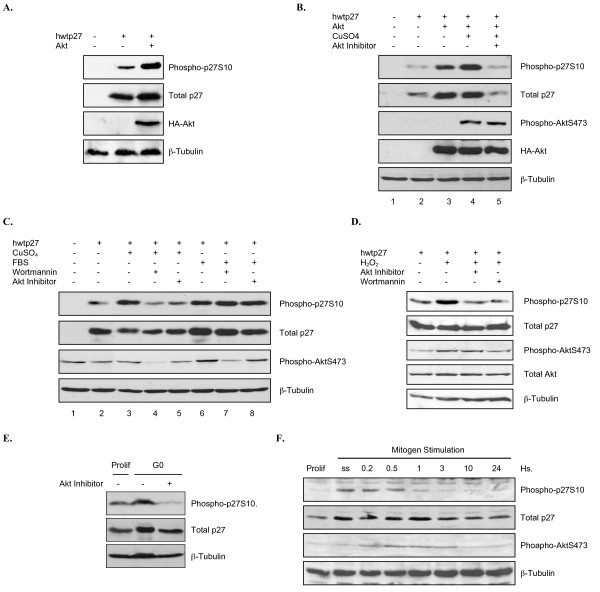
**Stress-activated Akt phosphorylates p27 at serine 10 in cells**. *A*, Overexpressed Akt1 phosphorylates S10 in HEK293 cells. hwtp27 was transiently overexpressed in HEK293 alone or in the presence of co-transfected Akt1. S10 phosphorylation was determined by western blot analysis after 36 hrs. *B*, CuSO_4_-dependent activation of Akt leads to p27S10 phosphorylation. Cells were transiently transfected as in A and treated with vehicle or CuSO_4 _for 2 hours prior to harvest. Samples were analyzed by western blot. Akt inhibitor was added 30 minutes prior to CuSO_4_. *C*, Endogenous Akt1 activated by oxidative stress phosphorylates p27S10. HEK293 cells were transfected with p27 and treated with CuSO_4 _or FBS for 2 hours prior to harvest. Inhibitors were added 30 minutes before treatment. Samples were analyzed by western blot. *D*, H_2_O_2_-activated endogenous Akt phosphorylates p27S10. Experiment was performed as in *C *treating the cells with H_2_O_2_. *E*, Growth factor withdrawal leads to an Akt-dependent phosphorylation of p27S10. Hela cells were grown in 10% FBS (prolif.) or serum starved (G0) in the presence or absence of Akt inhibitor. Cells were harvested and phosphorylation of S10 analyzed by western blot. *F*, Mitogen-activated Akt does not target p27S10. HDF cells were synchronized in G0 by serum deprivation and re-fed with 20% FBS. Cells were lysed at the indicated time points and analyzed by western blot.

Given the strong evidence that Akt1 phosphorylates p27 at S10 in vitro, we sought to determine if Akt1 also targets this site in cells. We first simply overexpressed p27 in HEK293 cells alone or co-transfected with Akt1. After 36 hours cells were harvested and analyzed for p27S10 phosphorylation by western blot. The amount of S10 phosphorylated p27 significantly increased in the presence of Akt1, consistent with our in vitro data indicating Akt1 targets this site (Figure 9A).

Akt is activated in response to a variety of different stimuli such as growth factors or cellular stress (50–53). Oxidative stress via exposure to copper was previously shown to activate Akt (50), so we examined whether treating cells with CuSO_4 _had an effect on p27 phosphorylation. Indeed, incubation of HEK293 cells overexpressing p27 and Akt1 with CuSO_4 _resulted in higher levels of p27S10 phosphorylation (Figure 9B, compare lanes 3 and 4). Total p27 levels also increased as expected given previous work showing S10 modification stabilizes p27 (28). Importantly, pre-treatment of cells with Akt inhibitor totally abolished S10 phosphorylation, indicating the CuSO_4 _effect was mediated by Akt1 (Figure 9B, lane 5). To determine if endogenous Akt targets p27S10 in response to stress, HEK293 cells were transfected with p27 alone and then treated with CuSO_4_. Exposure to CuSO_4 _again caused an increase in p27S10 phosphorylation (Figure 9C, compare lanes 2 and 3) which was abolished by pre-treating cells with wortmannin or Akt inhibitor (Figure 9C, compare lane 3 to lanes 4 and 5). As expected we observed increased levels of total p27 due to the increased stability of the S10 phosphorylated form (Figure 9C, middle panel).

To confirm that CuSO_4 _treatment was activating Akt1 via generation of reactive oxygen species (ROS), we repeated the experiment with H_2_O_2 _(51). H_2_O_2 _treatment of HEK293 cells overexpressing p27 with increased p27S10 phosphorylation, which was inhibited by pre-incubating cells with wortmannin or Akt inhibitor (Figure 9D). In marked contrast, mitogen stimulation also leads to increased Akt1 activation and p27S10 phosphorylation (Figure 9C). However, in this case it is not prevented by the Akt inhibitor or wortmannin. These results suggest Akt1 specifically phosphorylates p27 at S10 in response to cellular stress, while a different kinase carries out this modification in response to mitogens.

Previous analysis of p27S10 phosphorylation in cells revealed it increases in response to the stress of mitogen withdrawal. To determine if Akt1 is responsible we analyzed p27 phospho-S10 levels after serum starving Hela cells in the absence or presence of Akt inhibitor. Serum starvation increases p27S10 phosphorylation, and this effect is dramatically blocked by Akt inhibitor (Figure 9E). These results strongly suggest Akt is largely responsible for phosphorylating p27S10 in response to mitogen removal. In contrast, mitogen stimulation of serum-starved cells also leads to Akt1 activation, but as predicted above this does not correlate with nor lead to increased p27S10 phosphorylation (Figure 9F). These results suggest that although Akt1 is activated in response to a variety of stimuli, it specifically phosphorylates p27S10 when cells are exposed to cellular stresses such as reactive oxygen species or growth factor deprivation.

## Discussion

Previous work has clearly demonstrated that Akt1 phosphorylates p27 [30]. Due to the multifunctionality and complex regulation of p27, determining the physiological significance of this event would be greatly facilitated by precisely identifying the targeted residue(s). Akt1 is a serine/threonine kinase, so there are 27 potential targets in human p27 (18 serines and 9 threonines). Several residues throughout the polypeptide chain have been postulated as Akt1 targets (S10, T157, T187, T198, or combinations thereof), based mainly on deletion and mutation analysis using purified proteins and commercial recombinant Akt1 [30,39-42]. This methodology has proven effective at identifying physiologically relevant phosphorylation sites in many different proteins, including p27. For instance, cyclin E-CDK2 phosphorylation of p27T187 was initially revealed by this approach [24]. However, its relative ease and simplicity compared to more labor intensive techniques like peptide mapping is tempered by potential indirect effects (e.g. mutating a binding rather than phosphorylation site).

Recent attention has focused on p27T157 because of its location within a putative RXRXXT^157 ^Akt1 consensus site located within the p27 NLS (Figure 1) [39-42]. T157 is not present in mouse p27, however, which is curious given that both p27 and the PI3K/Akt1 pathway are highly conserved in the two species. This lack of conservation is in marked contrast to other physiologically relevant p27 phosphorylation sites such as T187 and S10. Nonetheless, there is precedence for nonconservation of sites with other Akt1 substrates. For example, Akt1 blocks apoptosis by phosphorylating human caspase-9 within the "RXRXXS^196^" consensus site even though S196 is not present in mouse and dog [44,45].

To try and resolve these issues we re-examined p27 phosphorylation by Akt1. Our results show that Akt1 phosphorylates human p27, mouse p27, and human p27T157A equally well. Phospho-peptide mapping of p27 revealed three radiolabeled spots, two of which lie in a diagonal characteristic of the same peptide containing different amounts of phosphate [43]. The less intense third spot may represent a partial digestion product that can arise when two basic amino acids (e.g. positions 15 and 19 in p27) are in the same vicinity [43]. Alternatively, the third spot might represent a less efficiently targeted residue residing on a different peptide. Phospho-amino acid and deletion analysis revealed that phosphate is incorporated almost exclusively into N-terminus serines. Our attention was immediately drawn to S10 for three reasons: 1) It is located in the only N-terminus tryptic peptide containing multiple serines (Table 1); 2) It has been previously identified as a major site of p27 phosphorylation in cells [28]; and 3) S10 was previously suggested as a potential Akt1 phosphorylation site [30]. A commercially available phospho-S10 antibody specifically recognized both mouse and human p27 after pre-incubation with Akt1 and ATP, confirming at least one of the modified residues is S10. Because phospho-peptide mapping indicated a second serine is located on the same peptide, it could be either S^7 ^or S^12^. Experiments are currently underway to precisely identify this site, which is complicated by its close proximity to S10 and the sequential nature of the reaction.

We generated hp27S10A with the expectation it would still be phosphorylated by Akt1 at the second site. However, the kinase completely failed to radiolabel this mutant, suggesting S10 modification is a pre-requisite for targeting the other residue. We attempted to mimic S10 phosphorylation by changing it to glutamic acid, but Akt1 did not label this mutant (data not shown). As an alternative approach we changed S10 to threonine. A kinase assay confirmed p27S10T is still phosphorylated, and phospho-amino acid analysis now revealed both radiolabeled threonine and serine. These results confirmed Akt1 phosphorylates S10, and showed its modification is required for targeting the second serine. Phospho-S10 could alter p27 structure to reveal the second site, perhaps involving the proline at position 11. Akt1's ability to sequentially target multiple residues in p27 raises the interesting possibility a similar mechanism occurs at the previously identified RXRXXS/T consensus site, which might have been overlooked by mutational analysis.

The region surrounding S10 is not the typical Akt1 consensus site (RXRXXS/T) originally characterized using a panel of purified peptides [46]. S10 is immediately adjacent to Pro11, and the peptide analysis suggested such a configuration is not targeted by Akt1 [46]. Obviously, however, the structure or recognition of an isolated peptide may be quite different within the context of a larger amino acid sequence. Thus, our results hint at the existence of a novel sequence motif that might be utilized in other Akt1 substrates. A blast search using a possible motif derived from the p27S10 region (RXSXXS/TPSXXR) identified several proteins involved in the PI3K/Akt1 pathway (e.g. Actin-binding LIM protein 1, PP2A, ABL1 and eIF4E binding protein). It will be of interest to determine if any of these potential candidates are phosphorylated by Akt1.

Why has there been so much confusion surrounding identity of the p27 residues(s) targeted by Akt1? One possible complication is pointed out by our observation that while Akt1 phosphorylates the N-terminus, it can also bind the C-terminus. Depending upon the experimental system and reaction conditions, mutations in this region could inhibit phosphorylation of the N-terminal residues. Such a situation is not unprecedented given that cyclin E-CDK2 binds p27 at the N-terminus region yet phosphorylates T187 [24]. Likewise, another candidate for S10 phosphorylation (hKIS) was originally identified by its affinity for the C-terminus portion of p27 [29]. The confusion could also be explained by the use of different recombinant Akt1 preparations, which we found display varying specificity towards potential substrates. These differences could arise due to a contaminating kinase, or more interestingly as a result of mutations introduced into Akt to facilitate its activation and/or purification. Regardless, our observations suggest caution is warranted when using recombinant Akt1 to characterize substrate specificity. For this reason and to confirm S10 is a relevant site, we analyzed p27 phosphorylation by full length wild type Akt1 in vitro and in cells. In both cases we found that Akt phosphorylates p27 at serine 10.

Many different stimuli activating Akt1 have been identified. We found that two well characterized types of cellular stress known to activate Akt1 – reactive oxygen species and growth factor withdrawal – also caused an increase in p27 phosphorylation at S10. Furthermore, pre-treating cells with small molecule inhibitors of Akt activation or activity blocked this stress dependent increase in S10 phosphorylation.

This effect appears to be quite specific, as mitogen stimulation also resulted in Akt1 activation but did not lead to an increase in S10 modification. Akt activation in response to stress is known to initiate a pro-survival response, and p27S10 phosphorylation/stabilization could play a role in this process by blocking cell cycle progression and/or modulating signal transduction pathways [18,19]. These possibilities must be tempered by our observation that when targeting S10 Akt1 can also phosphorylate a neighboring serine, which could have unexpected consequences or alter regulatory events typically associated with S10 modification. Given our in vitro analysis suggesting Akt1 specificity can vary, it remains possible that Akt1 targets p27 residues other than/in addition to S10 in response to different physiological conditions. Regardless, our observation that Akt1 phosphorylates p27S10 in response to cellular stress has important implications for current efforts to understand how PI3K/Akt1 normally regulates p27, as well as how this process is disrupted in human cancers.

## Conclusion

Akt1 phosphorylates p27 to regulate its activity, but the identity of targeted residue(s) remains controversial. Here we show that full-length wild type Akt1 – either purified or in cells – phosphorylates p27 at S10, a noncanonical but evolutionarily conserved site known to regulate p27 function. Using recombinant Akt1 that recapitulates this specificity, we showed modification of p27S10 also leads to phosphorylation of an adjacent serine. Although Akt1 is activated by many different stimuli, we found that it specifically phosphorylates p27S10 in response to cellular stress such as reactive oxygen species or growth factor deprivation. These results integrate PI3K/Akt1 signaling with p27 regulation through its major phosphorylation site in cells, and thus identify new avenues for understanding its deregulation in human cancers.

## Methods

### Protein expression constructs

P27, His-tagged Akt1, His-tagged cyclin E and CDK2 were cloned into the CS2+ mammalian expression vector in which protein production is driven by the CMV promoter [24]. HA-tagged Akt1 was a gift of Dr. Richard Nho. Tags are located at the N-terminus. For bacterial expression p27 was cloned into a Pet16B vector [24]. P27 mutants were generated using the QuickChange site-directed mutagenesis kit (Stratagene, La Jolla, CA) and confirmed by DNA sequencing.

### Recombinant proteins and protein purification

Purified GSK protein was purchased from Cell Signaling Technology. Purified recombinant Akt1 protein was obtained from Cell Signaling Technology (prep1:#7502; and prep3: #9274) and from Calbiochem (prep2). His-tagged p27 was purified from E. coli extracts by Ni^2+^-affinity chromatography. Briefly, the BL21 strain of E. coli was transformed with Pet16B expression vectors containing the cDNA of interest, grown to an OD of 0.6, and protein synthesis induced by 1 mM IPTG for 4 hours. Cells where lysed in non-denaturing buffer pH8 (50 mM NaH_2_PO_4_, 300 mM NaCl, 10 mM Imidazole, 0.05% tween) and His-tagged proteins purified under native conditions according to the manufacturer's directions using Ni-NTA kit (QIAGEN). Purified proteins were dialyzed against 10 mM Tris pH8.0, 1 mM EDTA, 20 mM NaCl, 2 mM DTT and 10% glycerol for 16 hours. P27 concentration was determined by the Bradford method and comparison with a known standard (BSA; BioRad) by Coomassie staining. His-tagged cyclin E-CDK2, full length His-tagged Akt1 and full length HA-tagged Akt1 were purified after transient overexpression in HEK293 cells. Cells were harvested under native conditions in non-denaturing buffer pH8. Full length His-tagged Akt1 and His-tagged cyclin E-CDK2 were purified by incubating whole-cell extract with Ni^2+^-agarose beads (QIAGEN) for 1 hour at 4°C. Beads were washed 3 times with 1 ml of the above lysis buffer containing 20 mM Imidazole, then once with kinase reaction buffer (25 mM Tris pH7.5, 5 mM β-glycerophosphate, 2 mM DTT, 50 nM Na_3_VO_3_, 5 mM MgCl_2_, and 0.2 mg/ml BSA). Full length HA-Akt1 was purified by immunoprecipitation with an antibody against HA-Tag (Santa Cruz, Santa Cruz, CA) using protein A-agarose (Roche) as described previously [22]. The kinase reaction was performed as described below.

### Kinase assays

Akt1 or cyclin E-CDK2 were incubated with purified His-tagged p27 in a final volume of 20 μL containing 20 μM non-labeled ATP (Sigma-Aldrich), 4 μCi [^32^P]-γ-ATP (ICM), and kinase reaction buffer (25 mM Tris pH7.5, 5 mM β-glycerophosphate, 2 mM DTT, 5 μM Na_3_VO_3_, 5 mM MgCl_2_, and 0.2 mg/ml BSA). Reactions were performed at 30°C for 60 min (unless otherwise indicated) and quenched by addition of SDS sampler buffer. Samples were heated at 90°C for 5 minutes, centrifuged at 13000 rpm for 3 minutes, and separated by SDS-PAGE. Proteins were then transferred to a PDVF membrane and incorporation of radiolabel visualized by autoradiography or phosphoimager.

### Phospho-peptide and phospho-amino acid analysis

Phospho-peptide (PPA) and phospho-amino acid (PAA) analysis were performed as described by Boyle et al. [43]. Briefly, kinase products were separated by SDS-PAGE and transferred to PDVF membrane. Bands containing the radiolabeled protein were visualized by autoradiography. For PPA, proteins were eluted by soaking excised membrane in 0.5% polyvinylpyrrolidone (PVP-360; Sigma) plus 100 mM acetic acid at 37°C for 30 minutes to block nonspecific absorption of trypsin. The membrane was then washed with water and 50 mM ammonium bicarbonate. Radiolabeled proteins were digested by incubation with 10 μg of trypsin in 50 mM ammonium bicarbonate at 37°C for 2 hours, followed by a second incubation with an additional 10 μg of trypsin overnight at 37°C. Phospho-peptides were spotted on cellulose-TLC plates and electrophoresed at 1500 V for 25 minutes. After air drying the plates were placed in a chromatography tank containing isobutyric acid buffer (1250 ml isobutyric acid, 38 ml n-Butanol, 96 ml pyridine, 58 ml glacial acetic acid, 558 ml water). Ascending chromatography was performed for 12 hours perpendicular to the direction of electrophoresis. Peptide maps were visualized by exposing plates to the phosphoimager. To determine identity of phosphorylated amino acids (PAA), bands containing radiolabeled proteins were hydrolyzed with 6N HCl at 100°C for 1 hour followed by lyophilization with a vacuum centrifuge. Dry pellets were resuspended in pH1.9 buffer containing 1 μg of nonlabeled phospho-serine and phospho-threonine standards. Because Akt1 is a serine threonine protein kinase it was not necessary to look for phospho-tyrosine, which allowed us to run electrophoresis in one dimension. Samples were spotted on TLC plates and electrophoresed at 1500 V for 25 minutes. Phospholabeled amino acids were visualized by exposing plates to the phosphoimager. Standards were visualized by 0.25% ninhydrin in acetone spraying and 15 minute incubation at 65°C.

### Cell culture

HEK293 and Human Diploid Fibroblasts (HDF) cells were obtained from ATCC and cultured in Dulbecco's Modified Eagle's Medium (DMEM) (GIBCO) supplemented with 10% (v/v) fetal bovine serum (FBS) (Atlas Biologicals) at 37°C in 5% CO_2_. Hela cells were obtained from Dr. Anja Bielinsky (University of Minnesota) and cultured in DMEM (1 mg/ml glucose) (Cellgro) supplemented with 10% (v/v) FBS (Atlas Biologicals). Mitogen deprivation was performed by growing cells to confluency and removing FBS for 48 hours. Cells were then stimulated with 20% FBS for the indicated times. When treating cells with CuSO_4_, DMEM was removed, cells were washed with PBS and incubated for 2 hours in Hanks' balanced salt solution (HBSS) containing CuSO_4_. Where indicated, cells were pre-treated with 200 nM wortmannin (Calbiochem) or 1 μM Akt inhibitor VIII (Calbiochem #124108) for 30 minutes, washed with PBS, and then incubated in HBSS containing CuSO_4 _and inhibitors.

### Transient transfections

Transfections were performed using the calcium-phosphate precipitation method as described previously [24]. Briefly, indicated amounts of plasmids containing the cDNA of interest were diluted in 500 μL calcium-phosphate solution, then added to 60 mm cell culture plates containing proliferating HEK293 cells in 3 mL of DMEM with 10% FBS. After 12 hours cells were washed with PBS and allowed to recover for 24 hours in DMEM plus 10% FBS prior to harvesting in indicated buffer. H_2_O_2 _was added to transfected cells for 2 hours prior to harvest. Where indicated, cells were pre-treated with 200 nM wortmannin (Calbiochem) or 1 μM Akt inhibitor VIII (Calbiochem #124108) for 30 minutes prior to H_2_O_2 _addition. In the case of CuSO_4 _treatment DMEM was removed, then cells were washed with PBS and incubated for 2 hours in Hanks' balanced salt solution (HBSS) containing CuSO_4_. Where indicated cells were pre-treated with 200 nM wortmannin (Calbiochem) or 1 μM Akt inhibitor VIII (Calbiochem #124108) for 30 minutes, washed with PBS, and then incubated in HBSS containing CuSO_4 _and inhibitors.

### Western blotting

Purified proteins or whole cell extracts were resolved by SDS-polyacrylamide gel electrophoresis (PAGE), transferred to PDVF membranes, and visualized by immunoblotting with indicated antibodies. Bound antibodies were detected using horseradish peroxidase-linked anti-mouse or anti-rabbit antibodies (Amersham Pharmacia Biotech), and visualized by enhanced chemiluminescence (Santa Cruz) followed by exposure to X-ray film (Kodak X-Omat AR). The antibodies employed are: anti-Akt1 and anti-phospho-AktS473 (Cell Signaling Technology); anti-p27 (Transduction Labs); anti-phospho-p27S10 and anti-HA-tag (Santa Cruz).

### Alkaline phosphatase treatment

After the kinase reaction utilizing non-labeled ATP, phosphorylated p27 was incubated for 1 hour at 30°C in the presence of 40 units of calf intestinal alkaline phosphatase (Promega) in 20 μL of buffer containing 50 mM Tris-HCl (pH 8.0) and 1 mM MgCl_2_. The reaction was stopped by adding SDS-sample buffer and then subjected to SDS-PAGE, followed by western blot analysis of total and S10 phosphorylated p27.

## Competing interests

The author(s) declare that they have no competing interests.

## Authors' contributions

LPN carried out protein purification, kinase assays, PPA, PAA, cloning and mutagenesis, and prepared the manuscript. RJS participated in cloning and mutagenesis, performed experiments in cells and prepared the manuscript. All authors read and approved the final manuscript.
